# Does degree of alteration in effort sense caused by eccentric exercise significantly affect initial exercise hyperpnea in humans?

**DOI:** 10.1186/s40101-016-0107-5

**Published:** 2016-08-24

**Authors:** Norio Hotta, Kaoru Yamamoto, Hisayoshi Ogata, Patrick Maher, Naoya Okumura, Koji Ishida

**Affiliations:** 1 College of Life and Health Sciences, Chubu University, Kasugai, Aichi 487-8501 Japan; 2 Faculty of Human Health Sciences, Meio University, Nago, Okinawa Japan; 3 Morioka Junior College, Department of International Cultural Studies, Iwate Prefectural University, Takizawa, Iwate Japan; 4 Graduate School of Life and Health Sciences, Chubu University, Kasugai, Aichi Japan; 5 Research Center of Health, Physical Fitness and Sports, Nagoya University, Nagoya, Aichi Japan

**Keywords:** Central command, Ventilation, Neuromuscular dysfunction, Delayed onset muscle soreness, Exercise onset

## Abstract

Previous research has shown an exaggeration in exercise hyperpnea 2 days after eccentric exercise (ECC). Enhancement in central command has been suggested as one candidate to account for this effect given that ECC-induced neuromuscular dysfunction increases relative exercise intensity, thus resulting in reinforcement of effort sense. The purpose of this study was, therefore, to elucidate whether the degree of alteration in effort sense caused by ECC affects exercise hyperpnea. Ten subjects performed 20-s single-arm extension-flexion exercises with weight strapped to the wrist, and ventilatory response was measured before (Pre) and 2 days after ECC (D2). Relative exercise intensity at Pre was 5 % of maximal voluntary contraction (MVC) of Pre, whereas that at D2 was 9 % MVC of D2 because of decline in muscle strength. Ventilatory responses were significantly exaggerated at D2 with a significant increase in effort sense. Although effort sense was significantly reduced during exercise at D2 when wrist weight was subtracted to match relative exercise intensity at Pre (5 % MVC of D2), ventilatory responses were still significantly higher than those of Pre. After the disappearance of post-ECC muscle damage, subjects performed the same exercise with weight added (9 % MVC of Pre) so that effort was equalized to match that of D2; however, no significant increase in ventilatory response was detected. The fact that the extent of change in effort sense caused by ECC-induced neuromuscular dysfunction did not affect ventilatory response at the onset of exercise after ECC may suggest that the exaggeration of ventilatory response after ECC is caused by mechanisms other than alteration of the central command.

## Introduction

Regulation of ventilatory response to physical activity is assumed to be controlled by neural and/or humoral mechanisms [1]. The neural controls are thought to consist of a “central command” [2] and afferent feedback signals via thin muscle afferents from exercising muscles [3]. Regarding central command, Bell [4] and Ward [5] explain in their reviews that it is one of the central neural mechanisms by which the central motor commands to contracting muscles provide a parallel stimulus to the brainstem respiratory integrating areas.

Exercise consisting of eccentric muscular work (ECC), in which muscle fibers contract while being stretched, is well known to induce loss of muscle strength and/or mechanical hyperalgesia or delayed onset muscle soreness (DOMS) [6]. The tendency for an exaggerated ventilatory response to exercise 2 days after ECC has been demonstrated by several studies [7–11]. Although the mechanisms underlying this ventilatory response have not been fully elucidated, prior research points to an enhancement in the central command as a candidate to account for this phenomenon [7, 9, 10]. The reason for this is because ECC-induced neuromuscular dysfunction increases relative exercise intensity, thus resulting in a reinforcement in central motor commands that subsequently increases effort sense [12], and many researchers presume that effort sense and central command might alter in parallel (e.g., [13, 14]).

In the present study, we aimed to reveal the effects of ECC-induced increases in relative exercise intensity or effort sense on exercise hyperpnea. To eliminate the effects of metabolic factors (e.g., chemoreflex and muscle metaboreflex) on ventilatory response and focus on neural mechanisms of ventilatory control (e.g., central command), we observed ventilatory responses at the onset of exercise (phase I) in the same way as in previous studies [15, 16].

## Methods

Eight men and two women [age 23.4 ± 5.4 years (mean ± SD), height 168.4 ± 7.0 cm, body mass 66.5 ± 8.6 kg] volunteered to take part in this study. All subjects provided written consent after being informed of the experimental protocol and potential risks involved in their participation. This study received the approval of the Human Research Committee of Research Center of Health, Physical Fitness and Sports of Nagoya University.

A single-arm flexion-extension exercise, the same as that used in our previous study [17], served as the basis for data collection and ventilatory response measurements. Briefly, subjects sat with a weight belt equivalent to 5 % of their maximal voluntary isometric elbow flexion (MVC) strength strapped to their wrist (regular weight trial). MVC strength was measured while seated with the elbow at 90° utilizing a load cell and a strain amplifier [17] or a muscle strength dynamometer. Each subject flexed and extended his/her elbow joint once per second for 20 s.

**Fig. 1:**
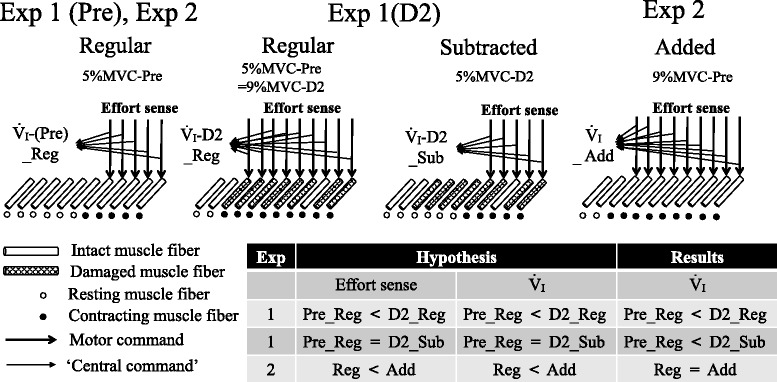
Rationale of the experimental design, hypothesis, and summarized results. For example, if maximal voluntary contraction (MVC) strength decreased from 20.0 kg [in which a 1.0-kg weight belt (5 % of 20.0 kg) was used during the regular weight trial (*Regular* or *Reg*) for measuring ventilatory response (*V.*_I_) before eccentric exercise (ECC) (Pre)] to 11.1 kg 2 days after ECC (D2) in experiment (Exp) 1, relative exercise intensity (weight belt/MVC strength) would increase to 9 % (1.0 kg/11.1 kg × 100) presumably with an increase in effort sense. The reason for this is that greater motor command outputs would be necessary to perform the regular weight trial because of reduced tension developments in muscle fibers damaged by ECC. Then, the relative exercise intensity would be expected to return to 5 % MVC during the subtracted weight trial (*Subtracted* or *Sub*) in which a 0.6-kg weight belt was used (5 % of 11.1 kg), and subsequently, effort sense would also be expected to return to the Pre level. In Exp 2, for instance, MVC strength is assumed to have already recovered from 11.1 to 20.0 kg. The ECC-induced increase in relative exercise intensity (9 % MVC = 1.0 kg/11.0 kg × 100) was reproduced by the added weight trial (*Added* or *Add*). In the trial, a 1.8-kg weight belt (9 % of 20 kg) would be utilized, conceivably resulting in the same level of effort sense as that in the regular weight trial at D2 without any influence from muscle damage and/or pain. As demonstrated by previous studies (e.g., [13, 14, 21]), effort sense could reflect the conscious awareness of the motor command sent to exercising muscles and effort sense and “central command” might be altered in parallel. Hence, we hypothesized that alteration in effort sense resulting from the change in relative exercise intensity caused by ECC-induced loss of muscle strength affects *V.*_I_

Subjects visited the laboratory four times. First, preliminary testing was carried out to familiarize the subjects with experimental procedures. The second visit involved measurement of ventilatory response to exercise (Pre), which was carried out just prior to subjects performing ECC in which the subjects completed three to six sets of 10 eccentric actions using a dumbbell set at 25–50 % of MVC strength [17]. During the third visit, 2 days after ECC (D2), experiment (Exp) 1 was carried out to examine whether exaggerated ventilatory response changes if the relative exercise intensity resulting from ECC-induced muscle strength loss was “subtracted” to match the Pre intensity level (subtracted weight trial). Experiment 2 was performed during the final visit, after the effects of ECC would have disappeared, thus allowing for examination of whether ventilatory response alters under undamaged muscle state when relative exercise intensity is “added” to match the D2 level of ECC-induced muscle strength loss (added weight trial). The rationale of the experimental design and our hypothesis are summarized in Fig. 1.

Each subject repeated the exercise four to eight times. Breath-by-breath inspiratory minute ventilation (*V.*_I_) data were aligned with the onset of exercise and then linearly interpolated between each breath to yield data at 1-s intervals [16]. Afterwards, ensemble averaging was carried out. As in our previous study [17], we defined the average for 30 s before the start of exercise as the resting value of *V.*_I_ (Rest) and the mean for the first 15 s of exercise as the exercising value of *V.*_I_ (EX).

A questionnaire for ratings of perceived exertion (RPE) to estimate the effort sense during exercise was used in previous studies (e.g., [13]). There was also a study that utilized RPE as a subjective index of central command [14]. We asked our subjects to indicate their RPE on the Borg scale [18] immediately after each exercise. We also evaluated the degree of muscle pain immediately after each exercise [17] using a visual analog scale (VAS) that had a 100-mm line with “no pain” on one end and “worst pain imaginable” on the other end [19]. The mean Borg scale ratings or VAS for each subject were then calculated and used as individually representative values.

A supplementary experiment was carried out for measurements in four additional healthy male volunteers (21.0 ± 1.4 years, 170.8 ± 6.1 cm, and 69.5 ± 9.9 kg) to confirm whether central motor drive and/or motor unit recruitment during the 20-s single-arm flexion-extension exercise varied between before and after ECC. The subjects performed the exercise five times. An electromyogram (EMG) was obtained from elbow flexor muscles using an active electrode and the signals were amplified, filtered (high pass 15 and low pass 500 Hz), and sampled at 1000 Hz. The sampled signals were full-wave rectified, averaged by each of the 20 elbow flexion-extension signals that were measured by a goniometer, and normalized to the EMG obtained during the MVC [20]. Ensemble averaging was carried out across five repetitions, and then, a mean of 20 averaged rectified values during the exercise was used as an individually representative value. The electrode position was marked so that it could be placed in a nearly identical spot before and after ECC.

For statistical analysis, we performed multivariate ANOVAs with repeated measures. Factors included “experiment” (Exp 1 and 2), “transition” (Rest and EX), and “trial” (regular, subtracted, and added weight trials). Factors were chosen in order to allow for particular comparisons. If a significant *F* value was observed in ANOVA, a Newman-Keuls post hoc test was used to identify specific differences. For paired-sample comparison, a paired *t* test was used for data that were normally distributed and a Wilcoxon signed-rank test was used for data that were not normally distributed. Statistical analyses were carried out using StatView 5.0 and SPSS 14.0 software, and the level of significance was set at 5 %. All values are expressed as means ± SE.

## Results and discussion

After ECC, MVC strength in all subjects declined from 27.1 ± 2.5 kg at Pre to 15.4 ± 1.5 kg at D2 (*P* < 0.0001). Additionally, muscle pain evaluations via a VAS showed that DOMS appeared in all subjects at D2 (from 0.0 ± 0.0 mm at Pre to 54.5 ± 8.0 mm at D2, *P* = 0.005).

**Fig. 2:**
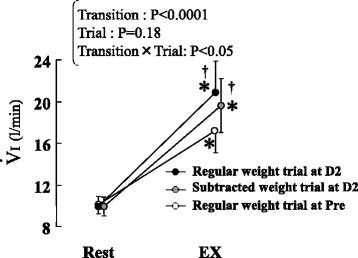
Change in inspiratory minute ventilation (*V.*_I_) from rest to the regular weight trials before (Pre) and 2 days after (D2) eccentric exercise (ECC) as well as the subtracted weight trial. The subtracted weight trial was performed at D2 and was arranged to compensate for the increase in exercise intensity attributed to ECC-induced loss of muscle strength during the regular weight trial at D2. The weight belts for the exercise were reduced so that the relative exercise intensity was equalized to that of the regular weight trial at Pre. The values of *V.*_I_ at Rest were the means for 30 s before the start of the trial. The values of *V.*_I_ at EX were the means for the first 15 s during each trial. Values are expressed as means ± SE (*n* = 10). *Asterisks* represent significant difference between Rest and EX (*P* < 0.05). *Daggers* represent significant difference compared to the regular weight trial at Pre (*P* < 0.05)

**Fig. 3:**
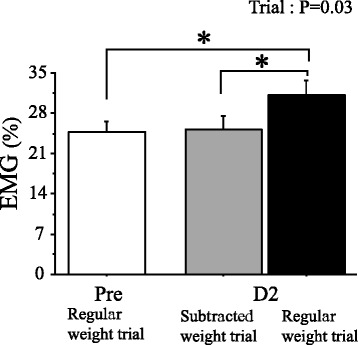
Changes in average electromyogram (EMG) values of elbow flexors during the regular weight trials before (Pre) and 2 days after (D2) eccentric exercise (ECC) as well as during the subtracted weight trial in the supplementary experiment (*n* = 4). In this additional experiment, after ECC, delayed onset muscle soreness (DOMS) appeared in all subjects (from 0.0 ± 0.0 mm at Pre to 31.5 ± 4.4 mm at D2, *P* = 0.006) and maximal voluntary contraction (MVC) strength significantly (*P* = 0.007) declined from 27.2 ± 1.0 to 15.6 ± 2.7 kg. For the regular weight trial, subjects’ wrists were fitted with weight belts (average weight = 1.3 kg) equivalent to 5 % of their MVC strength in *Pre*, and thus, the relative exercise intensity changed to 9 % at D2 due to the ECC-induced reduction in MVC strength. For the subtracted weight trial, weight belts equivalent to 5 % of their MVC strength in *D2* were used (average weight = 0.7 kg). Ratings of perceived exertion (RPE) during the regular weight trials was significantly (*P* < 0.05) increased from Pre (9.9 ± 0.9) to D2 (12.6 ± 1.4); however, RPE during the subtracted weight trial (10.2 ± 1.6) significantly (*P* < 0.05) declined compared with that during the regular weight trials at D2. Values are expressed as means ± SE. *Asterisks* represent significant difference (*P* < 0.05)

Data were collected during exercise sessions in which subjects’ wrists were fitted with weight belts equivalent to 5 % of their MVC strength at Pre (average weight = 1.4 kg). Reflecting previous studies [7–11], a significant increase in *V.*_I_ response was detected at D2 compared with that at Pre (*P* < 0.05) (Fig. 2); however, relative exercise intensity increased at D2 because the same weight belt used at Pre [average weight = 1.4 kg (9 % MVC of D2)] was utilized despite the fact that the muscle strength declined. In fact, the EMG signal (Fig. 3) and RPE (from 11.1 ± 0.7 to 13.9 ± 0.9) at D2 during the exercise significantly (*P* < 0.05) increased compared with those at Pre, being in accordance with the results of prior studies [7–9, 20]. It stands to reason that an enhancement in the central command might lie behind this phenomenon.

Immediately after initial data collection, the “subtracted weight trial” was arranged at D2. In that trial, exercise intensity was correlated to 5 % of the MVC obtained at D2 by subtracting weight from the belt (average weight = 0.8 kg). This resulted in a significant (*P* < 0.05) decline in the EMG signal (Fig. 3) and RPE (12.2 ± 0.8) during the subtracted weight trial compared with that in the regular weight trial at D2. Nevertheless, *V.*_I_ at EX was significantly (*P* < 0.05) higher than that in the regular weight trial at Pre, though no significant difference in *V.*_I_ was detected between the subtracted weight trial and the regular weight trial at D2 (Fig. 2). Therefore, Exp 1 demonstrates that significant exaggeration in initial exercise hyperpnea occurred after ECC regardless of ECC-induced augmentations in relative exercise intensity or effort sense.

**Fig. 4:**
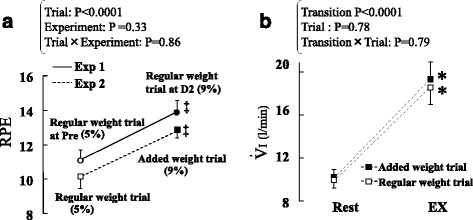
**a** Change in ratings of perceived exertion (RPE) in the regular weight trials before (Pre) and 2 days after (D2) eccentric exercise (ECC) in experiment (Exp) 1 as well as from the regular weight trials to the added weight trial in Exp 2 performed after subjects had recovered from ECC-induced muscle damage. The added weight trial was arranged to simulate the increase in exercise intensity during the regular weight trials at D2. *Double daggers* represent significant difference between trials in each Exp (*P* < 0.05). Percentages in parentheses show the relative weight for exercise to maximal muscle strength. **b** Change in inspiratory minute ventilation (*V.*_I_) from rest to the regular weight trials and the added weight trials. Abbreviations are the same as those in Fig. 2. Values are expressed as means ± SE (*n* = 10)

Shifting perspectives, to elucidate whether the extent of ECC-induced increases in relative exercise intensity and subsequent increases in effort sense per se affect initial exercise hyperpnea regardless of muscle state, Exp 2 was carried out under the condition in which the effects of ECC were absent. After the effects of ECC in Exp 1 had disappeared, subjects performed the following two types of exercise in random order. In exercise 1, each subject repeated the regular weight trial [average weight = 1.4 kg (5 % MVC of Pre)] as described earlier. In exercise 2, i.e., the “added weight trial,” additional weight belts were bound to the subject’s wrist to replicate an augmented relative intensity equal to that of the exercise at D2, in which MVC strength had decreased [average weight = 2.4 kg (average 9 % MVC of Pre; note that this value is the same as that of D2 in Exp 1)]. Figure 4a shows the changes in RPE. With no interaction between “trial” and “experiment,” the added weight trial in Exp 2 should have simulated the change in effort sense seen during the exercise at D2 in Exp 1.

Figure 4b shows the effects of the added weight trial on ventilatory response. There is no interaction between “trial” and “transition,” implying that the added weight trial did not have any significant influence on ventilatory response at the onset of exercise. Hence, Exp 2 demonstrates that the degree of change in relative exercise intensity and effort sense influenced by ECC did not significantly affect the ventilatory response at the onset of exercise. The rationale and results of this study are summarized in Fig. 1.

## Conclusion

Contrary to our hypothesis (Fig. 1), the degree of the alteration in effort sense resulting from the change in relative exercise intensity caused by ECC-induced loss of muscle strength did not significantly affect initial exercise hyperpnea. On the rationale that effort sense and central command might be altered in parallel (e.g., [13, 14]), the results may suggest that post-ECC muscle condition changes the central command during exercise but that at least the extent of the change does not significantly relate to an exaggeration in ventilatory response at the onset of exercise after ECC.

## Abbreviations

ECC: Eccentric muscular work or eccentric exercise
DOMS: Delayed onset muscle soreness
Pre: Before eccentric muscular work or eccentric exercise
D2: 2 days after eccentric muscular work or eccentric exercise
Exp: Experiment
MVC: Maximal voluntary contraction
*V.*_I_: Inspiratory minute ventilation
Rest: The resting value of inspiratory minute ventilation
EX: The exercising value of inspiratory minute ventilation
RPE: Ratings of perceived exertion
VAS: Visual analog scale
EMG: Electromyogram
